# Remnant cholesterol inflammatory index and chronic lung disease in middle-aged and older Chinese adults: A cross-sectional analysis of the China Health and Retirement Longitudinal Study

**DOI:** 10.1097/MD.0000000000048926

**Published:** 2026-05-29

**Authors:** Jian Liu, Xiangchen Guan, Xinlong Pang, Xiangyan Liu, Zhongwei Xin, Mo Shi

**Affiliations:** a Department of Thoracic Surgery, Shandong Provincial Hospital Affiliated to Shandong First Medical University, Jinan, Shandong, China; b Department of Thoracic Surgery, Pingyi County People’s Hospital, Linyi, Shandong, China.

**Keywords:** China Health and Retirement Longitudinal Study, chronic lung disease, high-sensitivity C-reactive protein, remnant cholesterol, remnant cholesterol inflammatory index

## Abstract

Chronic lung diseases are common among middle-aged and older adults in China. Because remnant cholesterol (RC) and systemic inflammation may jointly contribute to respiratory disease risk, simple blood-based composite markers may help identify individuals at higher risk. We conducted a cross-sectional analysis of the China Health and Retirement Longitudinal Study using biomarker data collected in 2011 and 2015. The study included 7694 adults aged ≥ 45 years with complete lipid and inflammation measurements at both time points. We calculated the RC inflammatory index (RCII), which integrates RC and high-sensitivity C-reactive protein, and constructed a time-weighted cumulative RCII between 2011 and 2015. Chronic lung disease was defined as a self-reported physician diagnosis of chronic bronchitis, emphysema, or chronic pulmonary heart disease. Multivariable logistic regression models were used to estimate associations. Overall, 968 participants (12.6%) reported chronic lung disease (CLD). Higher baseline RCII was associated with higher odds of CLD, with a dose–response pattern across quartiles. In the fully adjusted model, the odds ratios (ORs) across increasing baseline RCII quartiles were 1.18 (95% confidence interval [CI]: 0.83–1.50), 1.32 (95% CI: 1.11–1.53), and 1.47 (95% CI: 1.15–1.79), with the lowest quartile as the reference. Higher cumulative RCII was also associated with CLD, with corresponding ORs of 1.24 (95% CI: 1.07–1.44), 1.52 (95% CI: 1.31–1.76), and 1.72 (95% CI: 1.45–2.05). Higher RCII was independently associated with CLD in middle-aged and older Chinese adults. Although the observed associations were modest in magnitude and the outcome relied on self-reported physician diagnoses, RCII may represent an accessible composite epidemiologic marker warranting further investigation; its value for individual-level risk stratification requires validation through studies incorporating calibration, reclassification, and decision-analytic assessment.

## 1. Introduction

Chronic lung diseases are a leading contributor to morbidity and healthcare utilization in China, and their impact is amplified by rapid population aging and accumulated lifetime exposure. Large-scale burden estimates have highlighted the substantial population-level impact of chronic respiratory diseases and persistent age- and sex-related disparities in China.^[1]^

Chronic lung diseases constitute a heterogeneous spectrum that includes chronic obstructive pulmonary disease (COPD), bronchiectasis and other chronic airway disorders, as well as interstitial lung diseases such as idiopathic pulmonary fibrosis.^[2–4]^ Among these conditions, COPD remains the most prevalent and contributes prominently to disability and economic costs in China.^[2,5]^ Despite improvements in prevention and management, effective and easily implementable biomarkers for early identification of individuals at higher risk for chronic lung disease (CLD) remain limited.

Remnant cholesterol (RC), calculated as total cholesterol (TC) minus the sum of high-density lipoprotein cholesterol (HDL-C) and low-density lipoprotein cholesterol (LDL-C), represents cholesterol carried by triglyceride-rich lipoprotein remnants.^[6–8]^ Accumulating evidence indicates that RC is not only a marker of dyslipidemia but is also linked to oxidative stress, endothelial dysfunction, and low-grade systemic inflammation.^[9–11]^ In parallel, high-sensitivity C-reactive protein (hs-CRP) is a well-established biomarker of systemic inflammation and has been associated with COPD activity, prognosis, and chronic respiratory risk in epidemiological and meta-analytic studies.^[12–15]^

Recently, remnant cholesterol inflammatory index (RCII), which integrates RC and hs-CRP, has emerged as a composite marker reflecting coupled metabolic and inflammatory states. Because remnant lipoprotein burden and systemic inflammation are biologically intertwined but not fully overlapping, a combined index may capture risk information that is incompletely reflected by either component alone. Previous population-based studies suggest that RCII is associated with adverse cardiovascular outcomes, stroke risk, and mortality.^[16–18]^ However, evidence regarding RCII and CLD is sparse, and it remains unclear whether baseline RCII and long-term cumulative exposure to elevated RCII are associated with CLD in middle-aged and older adults.

Therefore, using China Health and Retirement Longitudinal Study (CHARLS) – a nationally representative longitudinal survey of Chinese adults aged 45 years and older initiated in 2011 to 2012 with follow-up waves in 2013, 2015, and 2018 – we aimed to evaluate the associations of baseline RCII and cumulative RCII exposure with CLD, compare RCII with its individual components (RC and hs-CRP), and explore potential non-linear dose–response relationships and effect modification in key subgroups.^[19]^ Notably, CHARLS collected and assayed venous blood biomarkers in both the national baseline wave (2011–2012) and the 2015 follow-up wave (Wave 3), enabling construction of RCII at multiple time points and assessment of longer-term exposure.^[20]^ We hypothesized that higher baseline and cumulative RCII would be associated with higher odds of CLD and that the combined index might better reflect the intertwined metabolic and inflammatory processes relevant to chronic respiratory disease.

## 2. Methods

### 2.1. Study design and data source

This study was a secondary analysis of CHARLS, a nationally representative ongoing longitudinal survey of community-dwelling Chinese adults aged 45 years and older. CHARLS applies a multistage, stratified probability sampling design and collects information using standardized questionnaires, physical examinations, and biomarker assessments; details of the cohort design and biomarker protocols have been described previously.^[20]^ This report was prepared with reference to the Strengthening the Reporting of Observational Studies in Epidemiology (STROBE) statement. The present analysis followed a cross-sectional design, relating baseline (2011) and time-weighted cumulative (2011–2015) biomarker indicators to CLD ascertained at the 2015 assessment.

CHARLS was approved by the Biomedical Ethics Review Committee of Peking University, and all participants provided written informed consent.^[20]^ Because the present analysis used de-identified public-use data, patients and the public were not involved in the design, conduct, reporting, or dissemination of this research.

**Figure 1.**
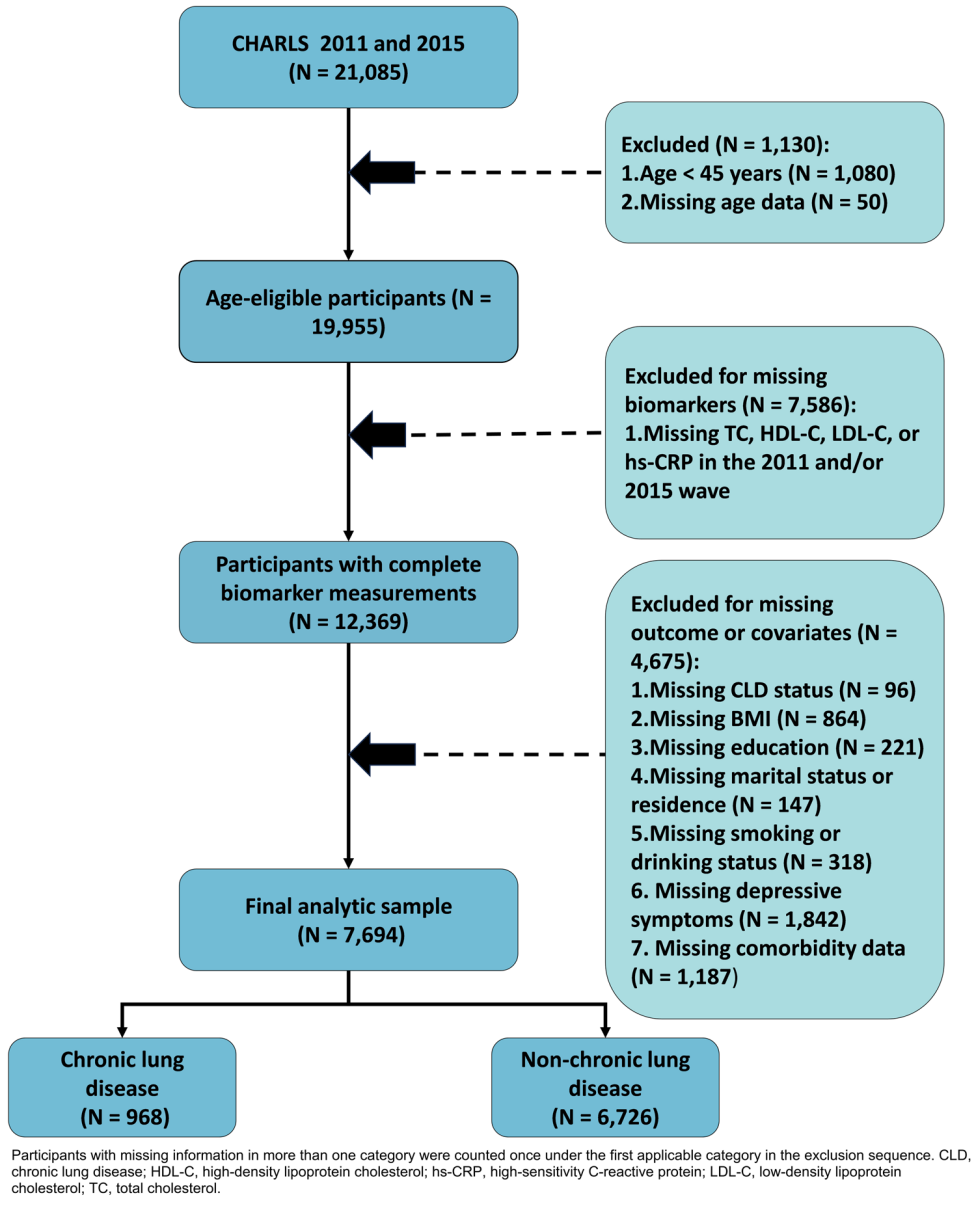
Flow diagram of participant selection from the CHARLS cohort. Counts for the third exclusion step are disaggregated by main variable category. CHARLS = China Health and Retirement Longitudinal Study.

For the current analysis, we used 2 CHARLS waves with venous blood biomarkers relevant to this study: the 2011 baseline survey (Wave 1) and the 2015 follow-up survey (Wave 3).^[20]^ Participant selection is summarized in Figure 1. Among individuals who participated in both the 2011 and 2015 surveys (n = 21,085), we first excluded those aged < 45 years (n = 1080) and those with missing age information (n = 50), yielding 19,955 age-eligible participants; we next excluded participants without measurements of TC, HDL-C, LDL-C, or hs-CRP in the 2011 and/or 2015 wave (n = 7586), leaving 12,369 participants with complete biomarker measurements at both time points; and finally, 4675 participants were excluded because of missing outcome or covariate information required for the multivariable models, comprising missing CLD status (n = 96), body mass index (BMI) (n = 864), educational attainment (n = 221), marital status or residence (n = 147), smoking or drinking status (n = 318), depressive symptoms (n = 1842), and comorbidity information covering hypertension, diabetes, dyslipidemia, cardiovascular disease, history of cancer, or history of liver disease (n = 1187), with participants having missing information in more than one category counted once under the first applicable category in the exclusion sequence so that category counts sum to the total excluded at this step. The final analytic sample comprised 7694 participants, of whom 968 reported CLD and 6726 did not.

### 2.2. Variables

Exposure assessment: Fasting venous blood samples were collected by trained medical professionals after at least a 12-hour overnight fast following standardized protocols.^[20]^ RC was calculated as RC = TC − (HDL-C + LDL-C) (mg/dL).^[6–8]^ RCII was calculated as RCII = [RC (mg/dL) × hs-CRP (mg/L)]/ 10. This composite index has been used in recent epidemiological studies examining stroke risk, mortality, and adverse cardiovascular outcomes.^[16–18]^

To characterize longer-term exposure between 2011 and 2015, cumulative indicators were computed using a time-weighted (trapezoidal) approach: cumX = [(X2011 + X2015)/2] × (2015 − 2011), where X denotes RCII, RC, or hs-CRP. For regression analyses, baseline and cumulative biomarkers were categorized into quartiles (Q1–Q4).

Outcome definition: Chronic lung disease was defined using the CHARLS health status questionnaire based on self-reported physician diagnoses. Participants were classified as having CLD if they answered “yes” to the item: “Has your doctor ever told you that you have chronic bronchitis, emphysema, or chronic pulmonary heart disease (excluding tumors or cancer)?” Objective measures such as spirometry, imaging-based adjudication, and subtype-specific clinical verification were not available in the public-use dataset used for this analysis; therefore, some outcome misclassification is possible.

Covariates: Potential confounders were selected a priori based on biological plausibility and previous studies. Baseline covariates were obtained from structured interviews and standardized measurements, including age (years), sex (male/female), residence (urban/rural), marital status, educational attainment (no formal education, primary school, junior high school, or high school and above), smoking status, drinking status, BMI, depressive symptoms, hypertension, diabetes, dyslipidemia, cardiovascular disease, history of cancer, and history of liver disease. Detailed smoking burden (e.g., pack-years), occupational exposures, medication use, and indoor or outdoor air-pollution variables were not consistently available in the analytic dataset and therefore could not be comprehensively incorporated into the primary models. Stroke, kidney disease, and gastrointestinal diseases were additionally considered in sensitivity analyses.

### 2.3. Statistical analysis

All analyses were performed using R software (version 4.4.3; R Foundation for Statistical Computing, Vienna, Austria). Continuous variables were assessed for distribution using the Shapiro–Wilk test (α = 0.10) and are presented as mean (standard deviation [SD]) for approximately normal distributions or median (interquartile range) for skewed distributions. Categorical variables are presented as counts (percentages). Group comparisons were performed using the chi-square test or Fisher exact test for categorical variables and the independent-samples *t* test or Mann–Whitney *U* test for continuous variables, as appropriate. Missing data were addressed using multiple imputation by chained equations with the mice package in R (version 3.16.0), under the assumption that data were missing at random. A total of 20 imputed datasets were generated, with 20 iterations per chain, and a fixed random seed was set to ensure reproducibility. The number of imputations (m = 20) was chosen to exceed the largest proportion of missingness across the variables entered into the imputation model, in line with current recommendations for multiple imputation. The imputation model included the exposure variables (baseline RCII and cumulative RCII, together with their constituent components RC and hs-CRP), the outcome (CLD), and all covariates entered into the regression models specified below. Predictive mean matching was used for continuous variables, logistic regression for binary variables, and multinomial logistic regression for categorical variables with more than 2 levels. Convergence was visually inspected using trace plots of the imputed values across iterations. Regression analyses were performed separately on each imputed dataset, and parameter estimates, standard errors, and 95% confidence intervals (CIs) were pooled using Rubin rules.

Associations of baseline RCII and cumulative RCII with CLD were evaluated using multivariable logistic regression, with odds ratios (ORs) and 95% CIs reported. RCII and cumulative RCII were modeled in quartiles (Q1–Q4), with Q1 as the reference category, and per SD increase. Models were adjusted sequentially as follows: Model 1 was unadjusted; Model 2 was adjusted for age and sex; and Model 3 was additionally adjusted for education, marital status, residence, smoking, drinking, BMI, depressive symptoms, hypertension, diabetes, dyslipidemia, cardiovascular disease, history of cancer, and history of liver disease. *P* for trend was calculated by modeling the median value of each quartile as an ordinal continuous term.

Restricted cubic spline regression was used to explore potential non-linear dose–response relationships for baseline and cumulative RCII. Prespecified subgroup analyses were conducted by fitting models within strata and testing multiplicative interaction terms. Receiver operating characteristic (ROC) curves and the area under the ROC curve (AUC) were used to assess the discriminative performance of the base model and the base model additionally including RC, hs-CRP, or RCII. All statistical tests were two-sided, with α = 0.05 indicating statistical significance.

### 2.4. Sensitivity analyses

Sensitivity analyses were conducted to evaluate the robustness of the main findings. We repeated the fully adjusted models after excluding participants with missing education or BMI and, separately, after excluding those with stroke, kidney disease, dyslipidemia, or gastrointestinal diseases.

## 3. Results

### 3.1. Participant characteristics

The analytic sample comprised 7694 participants, of whom 968 (12.6%) reported CLD. Baseline characteristics according to quartiles of baseline RCII are shown in Table 1. Across increasing RCII quartiles, hs-CRP and RC increased, TC and LDL-C increased, and HDL-C decreased (all *P* < .001). The distribution of BMI categories differed across quartiles (*P* < .001), and the proportions of kidney disease and gastrointestinal diseases also differed across quartiles (both *P* < .001) (Table 1).

**Table 1:** Baseline characteristics of participants according to quartiles of the remnant cholesterol inflammatory index.

Characteristic	Remnant cholesterol inflammatory index, quartiles	RCII	RCII	RCII	*P* for trend	*P*
	Q1 (n = 1922)	Q2 (n = 1922)	Q3 (n = 1922)	Q4 (n = 1922)	*P* for trend	N/A
Age, mean SD, yr	58.7 (8.9)	58.4 (8.8)	59.0 (8.8)	58.4 (8.9)	58.9 (8.9)	<.001
Gender(male), n (%)	4208 (50.7)	1014 (51.3)	1049 (50.2)	1093 (48.2)	1052 (51.2)	.913
Residence (urban), n (%)	2587 (33.6)	588 (35.2)	610 (29.3)	670 (34.2)	719 (33.7)	.940
Education, n (%)	7689	1929	1923	1925	1922	.032
Illiteracy	3887(50.6)	977 (51.7)	1013 (52.7)	927 (48.2)	970 (50.4)	N/A
Primary school	1632 (21.2)	412 (21.4)	387 (20.1)	412 (21.4)	421 (21.4)	N/A
Junior high school	1482 (19.3)	352 (18.2)	371 (19.2)	395 (20.6)	364 (18.9)	N/A
High school or above	693 (9.0)	183 (9.4)	152 (7.9)	182 (9.5)	176 (9.1)	N/A
BMI, n (%), kg/m^2^	7689	1924	1923	1925	1917	<.001
<18.0	304 (3.9)	96 (4.9)	94 (4.9)	59 (3.1)	55 (2.8)	N/A
18.0–24.0	3901 (40.2)	1216 (63.2)	1012 (53.0)	869 (45.1)	804 (41.6)	N/A
≥24.0	3481 (45.3)	612 (31.8)	814 (42.3)	997 (51.8)	1058 (54.9)	N/A
Comorbidities						
Hypertension, n (%)	2608 (33.9)	549 (31.2)	607 (32.2)	692 (34.8)	760 (34.2)	.112
Dyslipidemia, n (%)	1415 (18.4)	261 (17.4)	344 (18.6)	373 (17.8)	437 (19.6)	.114
Diabetes, n (%)	730 (9.5)	131 (9.1)	167 (9.7)	191 (7.8)	241 (8.5)	.986
Stroke, n (%)	271 (3.5)	46 (3.8)	80 (2.7)	62 (4.0)	83 (2.5)	.539
Kidney disease, n (%)	563 (7.3)	197 (5.7)	180 (6.3)	175 (7.9)	181 (10.8)	<.001
Gastrointestinal Diseases, n (%)	2439 (31.7)	647 (25.3)	602 (29.8)	584 (33.4)	606 (35.8)	<.001
Laboratory values						
MCV, 10^3^/μL	91.6 [88.3, 95.6]	92.0 [88.9, 96.6]	91.7 [88.4, 96.1]	91.5 [88.0, 95.3]	91.6 [88.3, 95.9]	.071
HDL-C, mean SD, mg/dL	51.5 11.6	54.1 12.0	52.2 11.4	50.2021 11.4	49.4 11.6	<.001
LDL-C, mean SD, mg/dL	104.5 29.0	101.4 26.6	103.0 28.1	105.2 29.5	107.4 31.4	<.001
Hs-CRP, median (IQR), mg/L	2.1 [0.8, 2.5]	0.8 [0.5, 1.0]	1.4 [0.9, 1.7]	2.2 [1.3, 2.7]	4.2 [1.8, 5.3]	<.001
CYSC, median (IQR), mg/L	0.9 [0.7, 1.0]	0.8 [0.7, 0.9]	0.9 [0.7, 1.0]	0.9 [0.7, 1.0]	0.9 [0.8, 1.0]	.121
TC, median (IQR), mg/dL	185.2 [160.6, 206.9]	177.8 [156.3, 197.6]	183.5 [160.2, 205.0]	187.2 [162.9, 209.3]	192.3 [165.6, 215.4]	.023
RC, median (IQR), mg/dL	30.1 [20.1, 35.9]	22.2 [16.9, 26.4]	26.3 [19.6, 31.6]	31.5 [22.9, 38.6]	40.4 [24.7, 51.3]	<.001
RCII	4.2 [0.9, 7.7]	0.6 [0.3, 0.8]	2.3 [1.6,3.1]	4.7 [3.8, 5.7]	10.7 [6.9, 18.8]	<.001

BMI = body mass index, CLD = chronic lung disease, HDL-C = high-density lipoprotein cholesterol, hs-CRP = high-sensitivity C-reactive protein, IQR = interquartile range, LDL-C = low-density lipoprotein cholesterol, RC = remnant cholesterol, RCII = remnant cholesterol inflammatory index, SD = standard deviation, TC = total cholesterol.

### 3.2. Associations of RCII with chronic lung disease

In the fully adjusted models (Model 3), higher baseline RCII was associated with higher odds of CLD (Table 2). Compared with Q1, the ORs were 1.18 (95% CI: 0.83–1.50) for Q2, 1.32 (95% CI: 1.11–1.53) for Q3, and 1.47 (95% CI: 1.15–1.79) for Q4; the OR per SD increase was 1.29 (95% CI: 1.20–1.38), with a significant trend across quartiles (*P* for trend < .001). Although statistically significant, the magnitude of the association was modest.

**Table 2:** Association between baseline remnant cholesterol inflammatory index and chronic lung disease.

Exposure	Group	Model 1, OR (95% CI)	Model 1, *P* for trend	Model 2, OR (95% CI)	Model 2, *P* for trend	Model 3, OR (95% CI)	Model 3, *P* for trend
	Group	OR (95% CI)	*P*	OR (95% CI)	*P*	OR (95% CI)	*P*
RCII	Q1	N/A	N/A	N/A	N/A	N/A	N/A
-	Q2	1.15 (1.03–1.28)	.022	1.09 (0.82–1.47)	.52	1.18 (0.83–1.50)	.452
-	Q3	1.38 (1.21–1.57)	<.001	1.42 (1.07–1.89)	.014	1.32 (1.11–1.53)	.008
-	Q4	1.55 (1.33–1.81)	<.001	1.45 (1.10–1.93)	<.001	1.47 (1.15–1.79)	<.001
Per SD increase	-	1.22 (1.14–1.31)	<.001	1.26 (1.16–1.36)	<.001	1.29 (1.20–1.38)	<.001

Model 1: unadjusted. Model 2: adjusted for age and sex. Model 3: adjusted for age, sex, education, marital status, residence, smoking, drinking, body mass index, depressive symptoms, hypertension, diabetes, dyslipidemia, cardiovascular disease, history of cancer, and history of liver disease.

CI = confidence interval, CLD = chronic lung disease, OR = odds ratio, RCII = remnant cholesterol inflammatory index, SD = standard deviation.

Higher cumulative RCII was also associated with CLD (Table 3). Compared with Q1, the ORs were 1.24 (95% CI: 1.07–1.44) for Q2, 1.52 (95% CI: 1.31–1.76) for Q3, and 1.72 (95% CI: 1.45–2.05) for Q4; the OR per SD increase was 1.30 (95% CI: 1.19–1.42), with a significant trend across quartiles (*P* for trend < .001).

**Table 3:** Association between cumulative remnant cholesterol inflammatory index and chronic lung disease.

Exposure	Group	Model 1, OR (95% CI)	Model 1, *P* for trend	Model 2, OR (95% CI)	Model 2, *P* for trend	Model 3, OR (95% CI)	Model 3, *P* for trend
	Group	OR (95% CI)	*P*	OR (95% CI)	*P*	OR (95% CI)	*P*
cumRCII	Q1	N/A	N/A	N/A	N/A	N/A	N/A
-	Q2	1.15 (1.03–1.28)	.013	1.20 (1.05–1.37)	.007	1.24 (1.07–1.44)	.004
-	Q3	1.38 (1.21–1.57)	<.001	1.45 (1.26–1.66)	<.001	1.52 (1.31–1.76)	<.001
-	Q4	1.55 (1.33–1.81)	<.001	1.63 (1.39–1.92)	<.001	1.72 (1.45–2.05)	<.001
Per SD increase	-	1.22 (1.14–1.31)	<.001	1.26 (1.17–1.36)	<.001	1.30 (1.19–1.42)	<.001

Model 1: unadjusted. Model 2: adjusted for age and sex. Model 3: adjusted for age, sex, education, marital status, residence, smoking, drinking, body mass index, depressive symptoms, hypertension, diabetes, dyslipidemia, cardiovascular disease, history of cancer, and history of liver disease.

CI = confidence interval, CLD = chronic lung disease, OR = odds ratio, RCII = remnant cholesterol inflammatory index, SD = standard deviation.

### 3.3. Discriminative performance of models including RC, hs-CRP, or RCII

**Figure 2.**
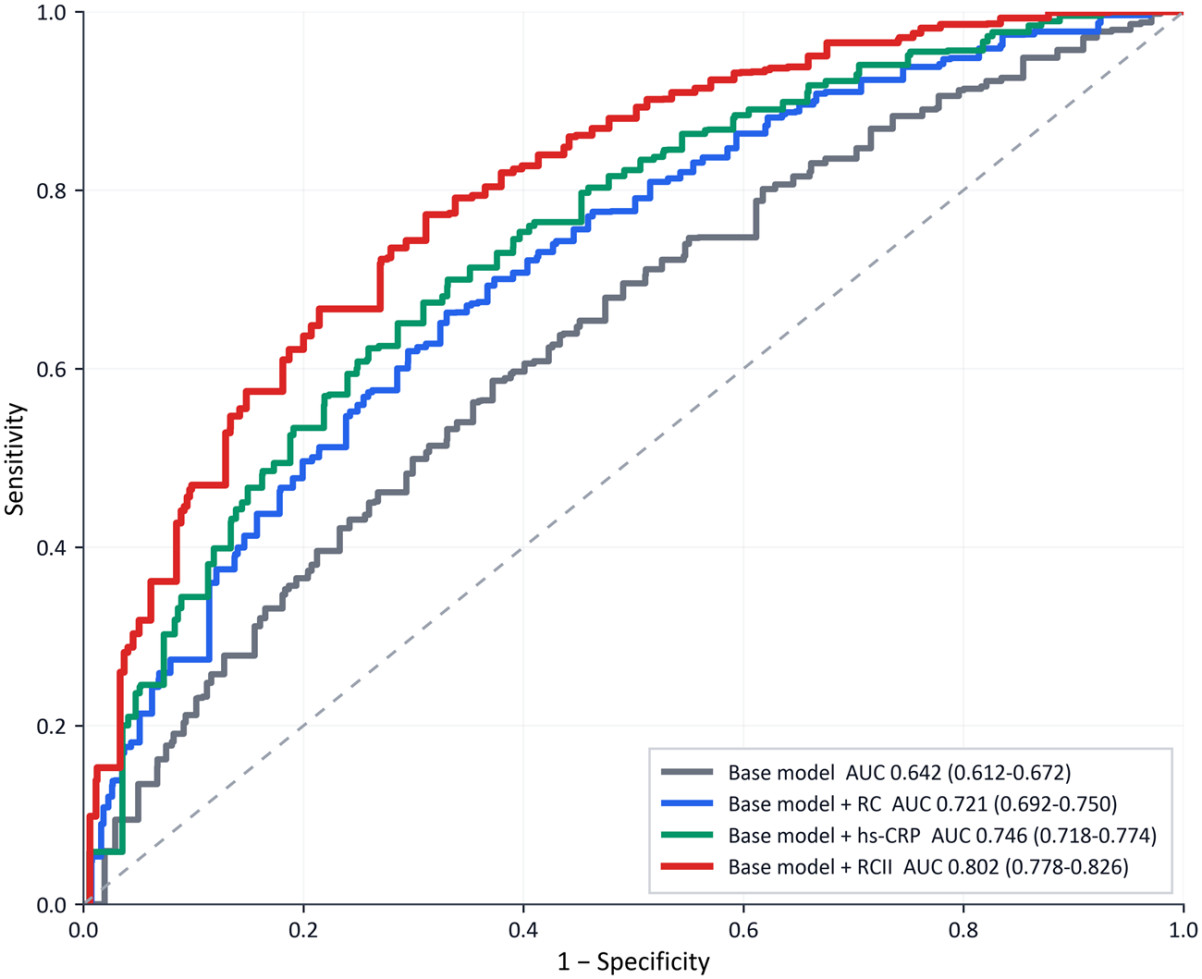
Receiver operating characteristic curves for the base model and models additionally including RC, hs-CRP, or RCII for chronic lung disease. Among the compared models, the model including RCII yielded the highest AUC. AUC reflects discrimination only; calibration, reclassification, and decision-curve analyses were not performed. hs-CRP = high-sensitivity C-reactive protein, RC = remnant cholesterol, RCII = remnant cholesterol inflammatory index.

ROC analysis was used to compare the discriminative performance of the base model and the base model additionally including RC, hs-CRP, or RCII (Fig. 2). The base model yielded an AUC of 0.642 (95% CI: 0.612–0.672), which increased to 0.721 (95% CI: 0.692–0.750) after adding RC, to 0.746 (95% CI: 0.718–0.774) after adding hs-CRP, and to 0.802 (95% CI: 0.778–0.826) after adding RCII. Among the compared models, the model including RCII yielded the highest AUC. Because model calibration, net reclassification improvement, integrated discrimination improvement (IDI), and decision-curve analysis were not performed, these results reflect discrimination only and should not be interpreted as evidence of improved individual-level risk prediction.

### 3.4. Sensitivity analyses

In sensitivity analyses excluding participants with missing education or BMI and, separately, those with stroke, kidney disease, dyslipidemia, or gastrointestinal diseases, the associations remained directionally consistent with the primary findings (Table 4). Across these analyses, the ORs for baseline RCII comparing Q4 with Q1 ranged from 1.40 to 1.53, and the ORs per SD increase ranged from 1.12 to 1.17. For cumulative RCII, the corresponding ORs ranged from 1.36 to 1.43 for Q4 versus Q1 and from 1.12 to 1.16 per SD increase, with all *P* for trend values remaining statistically significant.

**Table 4:** Sensitivity analyses of baseline and cumulative remnant cholesterol inflammatory index in relation to chronic lung disease.

Sensitivity analysis	N	CLD, n (%)	Baseline RCII: Q4 vs Q1, OR (95% CI)	Baseline RCII: per SD, OR (95% CI)	Baseline RCII: *P* for trend	Cumulative RCII: Q4 vs Q1, OR (95% CI)	Cumulative RCII: per SD, OR (95% CI)	Cumulative RCII: *P* for trend
Primary analysis (MI; Model 3*)	7694	968 (12.6)	1.55 (1.28–1.88)	1.18 (1.08–1.29)	<.001	1.44 (1.19–1.75)	1.16 (1.07–1.26)	.001
Complete-case (education & BMI non-missing)	7689	967 (12.6)	1.53 (1.26–1.87)	1.17 (1.07–1.29)	<.001	1.43 (1.18–1.74)	1.16 (1.06–1.26)	.001
Excluding stroke	7423	905 (12.2)	1.52 (1.24–1.86)	1.17 (1.07–1.29)	.001	1.41 (1.15–1.73)	1.15 (1.06–1.26)	.003
Excluding kidney disease	7131	842 (11.8)	1.48 (1.19–1.84)	1.16 (1.05–1.29)	.003	1.40 (1.12–1.75)	1.14 (1.04–1.25)	.007
Excluding dyslipidemia	6279	750 (11.9)	1.40 (1.10–1.78)	1.12 (1.01–1.25)	.021	1.36 (1.07–1.73)	1.12 (1.02–1.23)	.026
Excluding gastrointestinal diseases	5255	575 (10.9)	1.47 (1.13–1.90)	1.15 (1.02–1.29)	.012	1.39 (1.05–1.83)	1.13 (1.01–1.26)	.041

All estimates are from fully adjusted models (Model 3).

CI = confidence interval, CLD = chronic lung disease, OR = odds ratio, RCII = remnant cholesterol inflammatory index, SD = standard deviation.

### 3.5. Restricted cubic spline analyses

**Figure 3.**
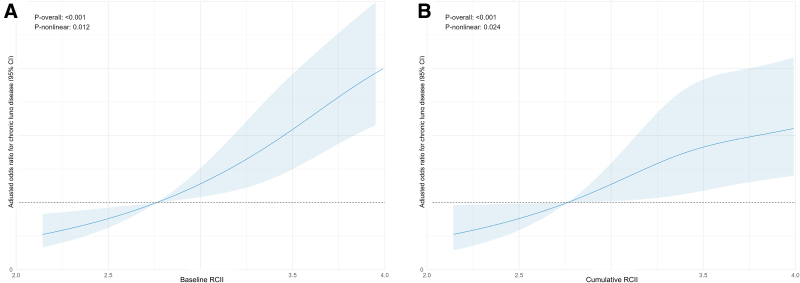
Restricted cubic spline associations of baseline (Panel A) and cumulative (Panel B) RCII with chronic lung disease. Solid lines denote adjusted odds ratios and shaded areas denote 95% confidence intervals. RCII = remnant cholesterol inflammatory index.

Restricted cubic spline models showed non-linear dose–response relationships for baseline RCII (*P*-overall < .001; *P*-nonlinear = .012) and cumulative RCII (*P*-overall < .001; *P*-nonlinear = .024) (Fig. 3).

### 3.6. Subgroup analyses

**Figure 4.**
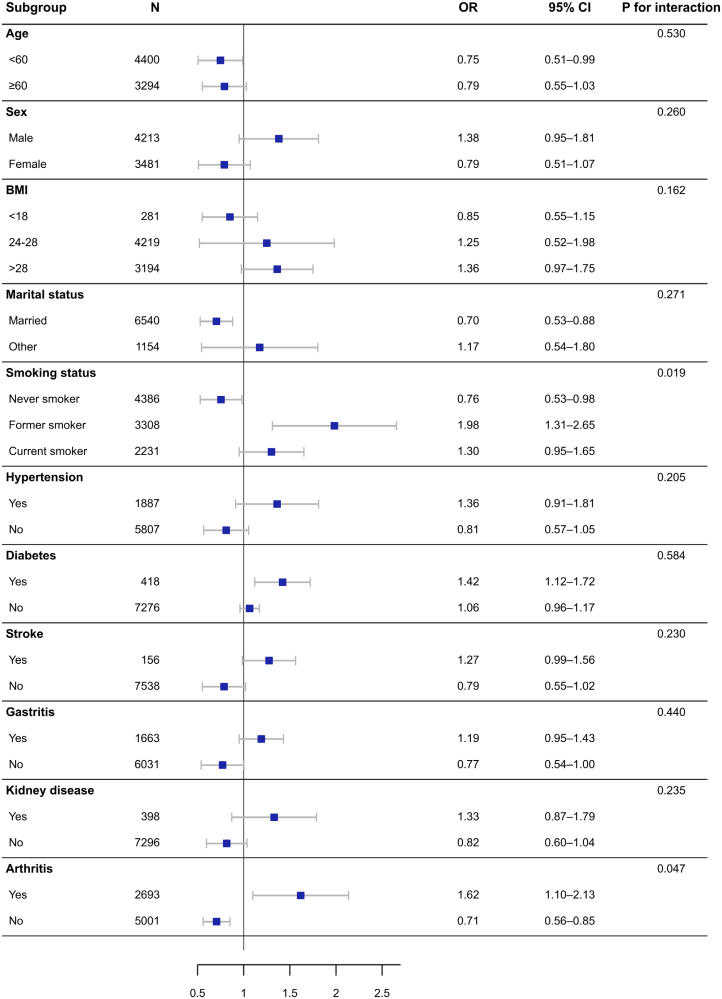
Subgroup analysis of the association between baseline RCII and chronic lung disease. Squares denote adjusted odds ratios and horizontal bars denote 95% confidence intervals. RCII = remnant cholesterol inflammatory index.

Subgroup-specific estimates are presented in Figure 4. Overall, the association between baseline RCII and CLD appeared broadly similar across most prespecified strata. Statistically significant interaction was observed for smoking status (*P* for interaction = .019) and arthritis (*P* for interaction = .047), whereas no significant interaction was observed for age, sex, BMI category, marital status, hypertension, diabetes, stroke, gastritis, or kidney disease (all *P* for interaction > .05).

For smoking status, the ORs were 0.76 (95% CI: 0.53–0.98) among never smokers, 1.98 (95% CI: 1.31–2.65) among former smokers, and 1.30 (95% CI: 0.95–1.65) among current smokers. For arthritis, the OR was 1.62 (95% CI: 1.10–2.13) in participants with arthritis and 0.71 (95% CI: 0.56–0.85) in those without arthritis. In the remaining subgroups, the corresponding estimates were 0.75 (95% CI: 0.51–0.99) in participants aged < 60 years and 0.79 (95% CI: 0.55–1.03) in those aged ≥ 60 years; 1.38 (95% CI: 0.95–1.81) in men and 0.79 (95% CI: 0.51–1.07) in women; 0.85 (95% CI: 0.55–1.15), 1.25 (95% CI: 0.52–1.98), and 1.36 (95% CI: 0.97–1.75) across BMI categories; 0.70 (95% CI: 0.53–0.88) in married participants and 1.17 (95% CI: 0.54–1.80) in others; 1.36 (95% CI: 0.91–1.81) in participants with hypertension and 0.81 (95% CI: 0.57–1.05) in those without hypertension; 1.42 (95% CI: 1.12–1.72) in participants with diabetes and 1.06 (95% CI: 0.96–1.17) in those without diabetes; 1.27 (95% CI: 0.99–1.56) in participants with stroke and 0.79 (95% CI: 0.55–1.02) in those without stroke; 1.19 (95% CI: 0.95–1.43) in participants with gastritis and 0.77 (95% CI: 0.54–1.00) in those without gastritis; and 1.33 (95% CI: 0.87–1.79) in participants with kidney disease and 0.82 (95% CI: 0.60–1.04) in those without kidney disease. Given the multiple subgroup comparisons and the reduced sample size within several strata, these findings should be interpreted cautiously and regarded as exploratory.

## 4. Discussion

In this nationally representative cross-sectional analysis of Chinese adults aged 45 years and older, higher baseline RCII was associated with higher odds of CLD after multivariable adjustment. Comparable associations were observed for the time-weighted cumulative RCII indicator derived from repeated biomarker measurements in 2011 and 2015. Risk estimates increased across higher RCII levels, and restricted cubic spline models further suggested non-linear dose–response relationships for both baseline and cumulative RCII. Although these associations were statistically significant, their magnitude was modest and should be interpreted cautiously in light of the observational design.

These findings are biologically plausible. RC reflects cholesterol carried by triglyceride-rich lipoprotein remnants and has been linked to endothelial dysfunction, oxidative stress, and inflammatory signaling.^[8–11,13,19]^ hs-CRP is a widely used marker of systemic inflammation and has been associated with COPD activity, prognosis, and chronic respiratory risk in epidemiological studies.^[12,14,21,22]^ RCII integrates these 2 dimensions and has shown prognostic value for cardiovascular outcomes, stroke, and mortality in population-based analyses.^[16–18]^ In the present study, ROC analysis showed that the model including RCII yielded a higher AUC than models additionally including RC or hs-CRP alone. This pattern is hypothesis-generating and is consistent with the possibility that a combined metabolic–inflammatory index captures complementary information beyond either component alone. However, AUC reflects discrimination only, and incremental predictive value cannot be established without assessment of model calibration, reclassification metrics (NRI and IDI), and decision-curve analysis, none of which was undertaken here. The apparent advantage of RCII observed in our data should therefore be regarded as preliminary and exploratory rather than as established evidence of improved risk stratification, and will require confirmation in studies that perform comprehensive predictive-performance evaluation.

Chronic lung disease, particularly COPD, is characterized by persistent airway and parenchymal inflammation with systemic inflammatory manifestations, and oxidative stress has been implicated as an amplifier of inflammatory responses and tissue injury.^[23–26]^ Against this background, the observed association between RCII and CLD is consistent with the hypothesis that coupled metabolic and inflammatory disturbances may contribute to chronic respiratory vulnerability. In addition, the cumulative RCII metric summarizes both the intensity and duration of exposure during 2011 to 2015 using a time-weighted approach, which may better reflect long-term biological burden than a single time-point measure. In other settings, repeated-measure indicators incorporating cumulative exposure have similarly been used to characterize long-term disease-related risk more comprehensively.^[22,27]^

In subgroup analyses, statistically significant interaction was observed for smoking status and arthritis, whereas interaction was not evident for most other participant characteristics. The stronger association observed among former smokers may reflect heterogeneity in cumulative inflammatory burden, smoking cessation history, medication use, or residual confounding across smoking categories. The interaction by arthritis status may likewise suggest differences in background systemic inflammatory activity. However, because multiple subgroup comparisons were performed and several strata had relatively small sample sizes, these findings should be regarded as exploratory and hypothesis-generating rather than definitive.

From a public health perspective, RCII can be derived from routinely available lipid fractions and hs-CRP levels without additional specialized testing. In population-based settings comparable to CHARLS, it may serve as an accessible epidemiologic marker of higher chronic respiratory risk. Nevertheless, the current evidence does not support its use as a standalone clinical decision-making tool or as a formal risk-prediction instrument. Instead, RCII may be more appropriately viewed as complementary to established risk factors and clinical assessment.

This study has several strengths. It used a nationally representative population-based dataset with standardized biomarker measurements at 2 time points, evaluated both baseline and cumulative exposure indicators, adjusted for a broad set of potential confounders, and yielded directionally consistent findings across multiple sensitivity analyses. Several limitations should also be acknowledged. First, CLD was defined on the basis of self-reported physician diagnosis, and spirometric confirmation and detailed phenotyping were not available, which may have introduced outcome misclassification. Second, the stepwise exclusion of participants with missing biomarker or covariate information may have introduced selection bias; however, the main categories of missing data and their per-category counts are transparently reported in Figure 1, and multiple imputation was applied within the analytic sample to address residual missingness under the assumption of missing at random, which should mitigate but cannot entirely exclude bias from this source. Third, the observational design precludes causal inference, and residual confounding from unmeasured or incompletely measured factors, including detailed smoking burden, occupational exposures, indoor or outdoor air pollution, medication use, and baseline lung function, cannot be excluded. Fourth, the outcome definition did not allow differentiation of specific CLD subtypes, such as COPD, bronchiectasis, or interstitial lung disease, and biomarker measurements at only 2 time points limited characterization of longer-term trajectories. Fifth, although ROC analysis suggested a higher AUC for the model including RCII than for models additionally including RC or hs-CRP alone, only discrimination was evaluated; model calibration, net reclassification improvement, IDI, and decision-curve analysis were not performed. AUC improvements alone do not establish clinical usefulness, and the apparent incremental value of RCII should therefore be interpreted as preliminary. Its utility for formal individual-level risk stratification requires comprehensive validation—including calibration assessment and decision-analytic evaluation—in independent populations.

Overall, the present findings suggest that higher baseline and cumulative RCII are associated with CLD in middle-aged and older Chinese adults. Further studies incorporating incident respiratory outcomes, objective lung function or imaging measures, more frequent biomarker assessments, and external validation populations are needed to clarify temporal relationships and better define the potential clinical and epidemiological utility of RCII.

## 5. Conclusion

Higher baseline and cumulative RCII were associated with CLD in middle-aged and older Chinese adults. Although the observed associations were modest in magnitude and the outcome relied on self-reported physician diagnoses, RCII may represent an accessible composite epidemiologic marker for hypothesis generation; its value for individual-level risk stratification remains to be established in studies incorporating calibration, reclassification, and decision-analytic evaluation. Further investigations incorporating objective respiratory outcomes, repeated biomarker assessments, and external validation populations are needed to clarify its potential clinical and public health relevance.

## Acknowledgments

We thank the National School of Development at Peking University for providing access to the CHARLS data .

## Author contributions

**Conceptualization:** Jian Liu.

**Data curation:** Jian Liu, Xiangchen Guan, Zhongwei Xin.

**Formal analysis:** Xiangyan Liu, Mo Shi.

**Funding acquisition:** Jian Liu, Mo Shi.

**Investigation:** Xiangchen Guan, Xiangyan Liu, Mo Shi.

**Methodology:** Jian Liu, Xiangchen Guan, Zhongwei Xin.

**Project administration:** Xinlong Pang, Mo Shi.

**Resources:** Jian Liu, Xiangchen Guan, Xinlong Pang, Mo Shi.

**Software:** Xiangchen Guan, Xiangyan Liu, Mo Shi.

**Validation:** Xiangchen Guan, Xinlong Pang, Xiangyan Liu, Mo Shi.

**Visualization:** Jian Liu, Zhongwei Xin.

**Writing – original draft:** Jian Liu, Xiangchen Guan.

**Writing – review & editing:** Jian Liu, Xiangchen Guan, Xinlong Pang, Xiangyan Liu, Zhongwei Xin, Mo Shi. All co-authors have reviewed and agreed to this revision.
